# Hospitals in New Zealand

**Published:** 1892-11-05

**Authors:** 


### COLONIAL HOSPITALS.

### HOSPITALS IN NEW ZEALAND.

Gentlemen upon whom the duty devolves of writing reports on the work of their departments might learn a good deal from a perusal of the report on the Hospitals and Charitable Institutions in New Zealand for the year 1892, as presented to the General Assembly by Dr. MacGregor, Inspector of Hospitals and Asylums in that colony. It is an able report, and a welcome contrast to the dry blue-books annually supplied by the head of the medical department in too many Governments with which we are acquainted.

This document is prefaced by an extremely interesting statement of the existing condition of the charitable institutions of New Zealand, and of the law by which they are regulated. According to Dr. MacGregor, there are two main ideas embodied in this law, viz.: (1) To decentralise administration as much as possible, and, at the same time, keep in check the evil tendency towards the multiplication of small local bodies inaugurated by the abolition of the provinces in 1876; and (2) to remedy the want of uniformity and justice in the distribution of subsidies to the different districts without at the same time drying up the springs of charity.

Dr. MacGregor regards decentralisation as a geographical and historical necessity, and as the chief characteristic of all the political and social problems in New Zealand. In 1870, it is true, a somewhat contradictory tendency was observed in the public works scheme then inaugurated, but although this may have been partly caused by an attempt to strengthen the central power against that of the provinces, it was really due to the necessity of consolidating the securities of the colony with a view to a great borrowing policy. In 1876 the provinces were abolished, without, however, any adequate provision being made for the devolution of the numerous local functions until then performed by them. The consequence was the successive creation of numerous local bodies armed with rating and borrowing powers, and when, in 1885, the attention of Parliament was compulsorily turned upon the hospital problem, it was found that the excessive number of local bodies, and the extinction of local charity, constituted two very grave dangers, against which some protection must forthwith be provided. While the multiplication of these local bodies was becoming an intolerable evil, local administration was yet recognized as being a vital necessity for the charitable institutions.

The first object of the Act of 1885, then, was to give effect to the prime necessity for local power based upon local taxation, and this was successfully achieved. Despite this advance, however, the whole system has been reduced to an absurdity by the excessive multiplication of local bodies, which has almost paralysed the working of the new law, and the lesson taught by the experience of the years since 1885 is that "contradictory tendencies cannot be successfully harnessed together even by a Parliament."

The second object of the Act of 1885 was to remedy the want of uniformity and justice in the distribution of charitable subsidies without at the same time drying up the springs of charity. Under the old system the province of Otago had long been distinguished for its generosity in voluntarily subscribing to the support of its charitable institutions, while the province of Canterbury was noted for the magnificence of its public grants. One feature of the voluntary system undoubtedly is that—to use Dr. MacGregor's words—"the willing and accessible few have to pay for the inaccessible and indifferent many." This happened in Otago. The people began to rebel against the voluntary system, and they were still further exasperated by the fact that the State grant in their case had only been £276 in 1884, whereas in Canterbury it had been £9,664, in addition to the sums given on the pound for pound principle, and that Auckland received £2,931 and Taranaki £850, although the voluntary subscriptions in that year had only amounted to £66.

Parliament admitted the injustice, and determined to systematise the distribution of subsidies, but, in view of the fact that the tax-gatherer is often the murderer of the voluntary subscriber, it was decided that for all subscriptions the State should give a subsidy of twenty-four shillings to the pound, while the rates should only be subsidised pound for pound. The following fact is significant. In 1884, the year before the Act was passed, Otago subscribed £3,242 voluntarily, and Canterbury £1,100. For 1892 Otago subscribed £203, and Canterbury £29. In a word, the systematisation of the distribution of subsidies has practically stopped all voluntary contributions.

We quote Dr. MacGregor again, as we cannot improve upon his terse epigrammatic language: "Law has commuted the voluntary burdens of the benevolent minority into a compulsory tax on all citizens; constraint has superseded duty; and who shall appraise correctly the loss and gain of such an exchange? We cannot escape the consequences of our neglect of social duty. Let this fail beyond a certain point, and, if society is to exist, compulsion must be brought in to support it. This despairing of duty, and this falling back on compulsion is illustrated in rather a startling fashion in our dealings with many other social problems. With regard to nearly all of them we seem to have been living in a fool's paradise of hope. Duty is considered to have become bankrupt in so many of our undertakings that there seems to be a world-wide movement in favour of compulsion by law."

"The New Zealand Hospitals and Charitable Institutions Act of 1885 has successfully localised administration and taxation, it has established a uniform system of distributing subsidies; but it has also fixed in the popular mind, and especially in the minds of the least self-reliant of our people, the belief that they have a right to a living whether they work or not."

Such is the present state of the problem. In a comprehensive Local Government Act Dr. MacGregor believes the ultimate solution will be found. He would consolidate the local bodies into about twenty strong Boards for the whole colony, and recast the entire system of subsidies in aid of local government. Into these suggestions and into some other points of his preface we shall not now inquire, contenting ourselves with this survey of the present situation. Evolution is a long process, and New Zealand is young. It bodes well for the future of her charitable institutions that she has not only a gentleman of Dr. MacGregor's acumen as Inspector of Hospitals and Asylums, but also a public sufficiently interested in their welfare to make it worth the inspector's while to write such a report as that which lies before us.

Referring briefly to the statistics with which the report concludes, we may add that 8,401 patients were treated in the 38 hospitals of the colony during the year ending on the 31st of March, 1892. Of these, 6,844 were discharged, 703 died, and 854 remained at the end of the year. The total collective days' stay in hospital was 301,841, and the individual average days' stay was 35 93. The average daily cost of each patient varied greatly in the various hospitals, being as high as £2 0s. 2d. at the Coromandel Hospital, and as low as 3s. 1¾d. at the Greymouth. At the former institution (which the inspector advises should be closed altogether) there were only ten patients during the year.

The total receipts of these 38 institutions were £79,189 6s.; of this sum £30,658 10s. 4d. came from Government, £23,559 15s. 3d. from hospital boards and local authorities, £4,736 6s. 7d. from voluntary contributions, £3,493 1s. 8d. from bequests, and £9,318 7s. 10d. from patients' payments. The rest accrued from rents, surplus from the previous year, and other sources not specified. On the other hand the total expenditure amounted to £74,039 4s. 7d., leaving a balance of £5,150 1s. 5d. towards the expenses this year. None of the statistics call for any special comment.

